# *Areca catechu*-(Betel-nut)-induced whole transcriptome changes in a human monocyte cell line that may have relevance to diabetes and obesity; a pilot study

**DOI:** 10.1186/s12902-021-00827-1

**Published:** 2021-08-14

**Authors:** Shirleny R Cardosa, B. William Ogunkolade, Rob Lowe, Emanuel Savage, Charles A Mein, Barbara J Boucher, Graham A Hitman

**Affiliations:** 1 Centre for Genomics and Child Health, Blizard Institute, Barts and the London School of Medicine and Dentistry, Queen Mary University of London, London, UK; 2 Barts and The London Genome Centre, Blizard Institute, Queen Mary University of London, London, UK

**Keywords:** Betel-nut, Type 2 diabetes, Obesity, Transcriptomics, RNA-sequencing

## Abstract

**Background:**

Betel-nut consumption is the fourth most common addictive habit globally and there is good evidence linking the habit to obesity, type 2 diabetes (T2D) and the metabolic syndrome. The aim of our pilot study was to identify gene expression relevant to obesity, T2D and the metabolic syndrome using a genome-wide transcriptomic approach in a human monocyte cell line incubated with arecoline and its nitrosated products.

**Results:**

The THP1 monocyte cell line was incubated separately with arecoline and 3-methylnitrosaminopropionaldehyde (MNPA) in triplicate for 24 h and pooled cDNA indexed paired-end libraries were sequenced (Illumina NextSeq 500). After incubation with arecoline and MNPA, 15 and 39 genes respectively had significant changes in their expression (*q* < 0.05, log fold change 1.5). Eighteen of those genes have reported associations with T2D and obesity in humans; of these genes there was most marked evidence for *CLEC10A*, *MAPK8IP1*, *NEGR1*, *NQ01* and *INHBE* genes.

**Conclusions:**

Our preliminary studies have identified a large number of genes relevant to obesity, T2D and metabolic syndrome whose expression was changed significantly in human TPH1 cells following incubation with betel-nut derived arecoline or with MNPA. These findings require validation by further cell-based work and investigation amongst betel-chewing communities.

**Supplementary Information:**

The online version contains supplementary material available at 10.1186/s12902-021-00827-1.

## Background

Obesity and T2D are reaching epidemic proportions worldwide, but particularly so in South Asian communities living in the Indian-subcontinent or who have migrated to other countries [1]. In the UK there is a three to four fold increase in T2D prevalence in South Asians compared to the general population; furthermore, the disease manifests at a 10–15 years younger age and is strongly associated with the metabolic syndrome and cardiovascular disease. Apart from lifestyle, potentially reversible environmental factors driving this disease are largely unknown.

Betel quid consumption is the fourth most common addictive habit, used by 10 % of the global population and very common in South Asians. The link between cancer risks (oropharyngeal, oesophageal and hepatocellular) and the ‘betel-chewing’ habit is well established [2–4]. Evidence also demonstrates associations between betel consumption and obesity, the metabolic syndrome and T2D. In a meta-analysis of 17 Asian studies, betel quid chewing was found to be significantly associated with obesity (relative risk (RR) = 1.47), metabolic syndrome (RR = 1.51), diabetes (RR = 1.47), hypertension (RR = 1.45) and cardiovascular disease (cardiovascular disease: RR = 1.2) [5]. Furthermore, in the Keelung Community Integrated Screening program from Taiwan, paternal betel-chewing was associated, dose-wise, with increases in early onset metabolic syndrome in their never-chewing offspring, while betel-chewing in adults increased their risks, dose-wise, of early onset T2D, cardiovascular disease and metabolic syndrome [6–8]. These data in humans support earlier data reported in CD1 mice, where it was found that a proportion of betel-fed adult mice developed hyperglycaemia and obesity and, most remarkably, that amongst their never-betel-fed offspring hyperglycaemia was detected in up to the 4th generation and that the vertical transmission of hyperglycaemia was associated with paternal, but not maternal, hyperglycaemia [9].

Betel quids (also known as paan) usually contain betel (*areca catechu*) nut, slaked lime and sometimes tobacco wrapped in *piper betle* leaf [10] though in Taiwan tobacco is not used. Nitrosation of the major arecal alkaloid, arecoline, generates MNPA and 3-methylnitrosaminopropionitrile (MNPN); both these compounds have been accepted as carcinogens [11]. Many nitroso-compounds have been reported as being diabetogenic. For instance, all reported survivors of poisoning by the nitroso-rat poison Vacor *(Pyrinuron (Pyriminil))* in the US have developed ketosis-prone insulin-dependent diabetes while ~ 20 % of survivors of Vacor poisoning in Korea developed ketosis-prone type 1 diabetes (T1D) but the other ~ 80 % developed non-insulin dependent diabetes; the difference being attributed to the lower content of the active ingredient in the Vacor packs sold in Korea than in those sold in America [12]. Furthermore, studies in mouse strains normally free of diabetes have regularly shown induction of ‘non-immune T1D’ following single large doses of nitroso compounds, classically streptozotocin (STZ) [13]. In contrast, repeated small doses of STZ induce a non-insulin dependent form of diabetes with enlarged pancreatic islets and beta cell abnormalities whose appearance matches those seen in pancreatic islets in humans developing T2D. With direct relevance to the current study, these same abnormalities were also seen in the pancreatic islets of the 8.3 % of young adult CD1 mice that developed hyperglycaemia with obesity following being fed standard RM1 rodent chow containing ground *Areca catechu* nuts for 2–6 days; interestingly, those changes also appeared in the islets of the 21.7 % of the never-betel-fed F1 offspring of the originally betel-fed mice that themselves developed obesity and hyperglycaemia [9].

These studies provides evidence that betel-chewing might be one of the aetiological factors for the increases in T2D and associated metabolic disorders in South Asians [12, 14]. Mechanisms that might link betel chewing with these disorders include inflammation, increases in hepatic synthesis of lipids and glucose, in adipogenesis, in adipose tissue glucose uptake, reductions in lipolysis and glycolysis, or neurological, hepatic or intestinal effects on appetite and adverse effects on vitamin D metabolism [15, 16].

In the present ‘proof of principle’ study we sought to investigate possible gene effects that might explain the links between T2D, obesity and related disorders and exposure to arecoline and its nitrosated products in a human monocyte derived cell line (THP1) using a whole transcriptome sequencing approach. THP1 was chosen due the central role that low-grade inflammation plays in the underlying causes and progression of obesity (for example through adipose tissue macrophages), metabolic syndrome and related disorders including both cardiovascular disease and T2D [17, 18]. It was also hoped to determine whether the strength of the evidence for any of the genes significantly affected might warrant further cell-based studies and investigation amongst betel-chewing communities.

## Results

### Determination of concentration of arecoline, MNPA and MNPN to be used in cell based assays

Cell viability using serial dilutions of arecoline extracts in culture media was assessed by phase contrast light microscopy. Representative images are seen in Additional Fig. 1. Control THP-1 cells are clear, round and uniform in shape, size and clarity. When THP1 cells are incubated for 48 h or more with arecoline concentrations of either 10 or 50 μg/ml the cells look healthy with similar regular appearance by phase contrast microscopy to controls. In contrast, cells incubated with 200 or 1000 μg/ml, appear unhealthy being shrunken and of irregular shape with loss of clarity and release of much disorganised cellular debris. No cell death or damage was observed in incubates containing MNPA or MNPN up to a concentration of 200 μg/ml, though at very high concentrations, media containing more than 1 % of the solvent (methanol and ethyl acetate) was toxic to the cells as assessed by phase light microscopy. A MTT assay for cell viability and proliferation was also performed (Additional Fig. 2), that demonstrates the THP-1 cells are healthy after incubation with a concentration of 50 μg/ml of arecoline but that toxicity was induced at 200 μg/ml and above, supporting the phase contrast light microscopy data.

### Selection of samples for whole transcriptome analysis

Whilst an inflammatory cellular response was seen after incubation of the THP1 cells with either arecoline, MNPA or Phorbol 12-myristate 13-acetate (PMA), minimal responses were found at 6, 24 or 48 h with MNPN and therefore, we did not proceed further with investigation of the last of these compounds (Additional Fig. 3).

PMA induces a macrophage-like phenotype differentiation in THP-1 cells, functionally mimicking many aspects of primary human macrophages. PMA, as a specific activator of Protein Kinase C, activates nuclear factor-kappa B and is regularly used as an internal control to test for cytokine and biological responsiveness of THP1 cells. In the absence of any response, one would have to conclude that data from the planned experiments on that particular cell line could not be used. We therefore decided to proceed with the incubations at 24 h. All transcriptome expression experiments (including whole transcriptome) were run in triplicate from independent rounds of cell culture analysis.

### Whole transcriptome analysis

#### Incubation with arecoline

Two hundred seventy-five gene hits were identified with a *q* < 0.05 (Additional Table 1) reducing to 15 with a log-fold change in either direction of 1.5 (Table 1). The relevance of these genes to diabetes, obesity and/or metabolic syndrome was assessed independently by 2 of us (BJB and GAH) with reference to GeneCards, The Type 2 Diabetes Knowledge Portal and PubMed as described in the methods. Four genes were identified of potential interest:

a Insulin Like Growth Factor Binding Protein 3 (*IGFB3* non-logged fold change 0.08),
b C-Type Lectin Domain Containing 10 A (*CLEC10A* fold change 0.14),
c Junction Plakoglobin (*JUP* fold change 0.21).
d Mitogen-Activated Protein Kinase 8 Interacting Protein 1 (*MAPK8IP1* fold change 0.21.

**Table 1:** Genes identified after incubation with arecoline

Gene Name	*q*-value^a^	Logfc^b^	Gene Description
*H3F3AP4*	0.03	-6.77	H3 Histone Pseudogene 6
*MEP1A*	0.04	-2.16	Meprin A Subunit Alpha
*KBTBD11-OT1*	0.05	-3.80	KBTBD11 Overlapping Transcript 1
*IGFBP3*	0.04	-2.28	Insulin Like Growth Factor Binding Protein 3
*AC097372.1*	0.04	-2.18	Reeler Domain Containing 1
*PTCRA*	0.04	-1.98	Pre T Cell Antigen Receptor Alpha
*IL3RA*	0.04	-1.96	Interleukin 3 Receptor Subunit Alpha
*CLEC10A*	0.03	-1.88	C-Type Lectin Domain Containing 10 A
*ACKR3*	0.04	-1.75	Atypical Chemokine Receptor 3
*JUP*	0.04	-1.50	Junction Plakoglobin
*MAPK8IP1*	0.05	-1.51	Mitogen-Activated Protein Kinase 8 Interacting Protein 1^b^
*MATK*	0.03	-1.51	Megakaryocyte-Associated Tyrosine Kinase
*TREML3P*	0.03	1.93	Triggering Receptor Expressed On Myeloid Cells Like 3, Pseudogene
*AC245036.5*	0.04	2.99	RNA gene; lncRNA
*AC113189.4*	0.05	2.75	RNA gene; lncRNA

Legend: Listed genes satisfied the following criteria *q* < 0.05^a^ and a log-fold change (logfc) of 1.5^b^

**Fig. 1:**
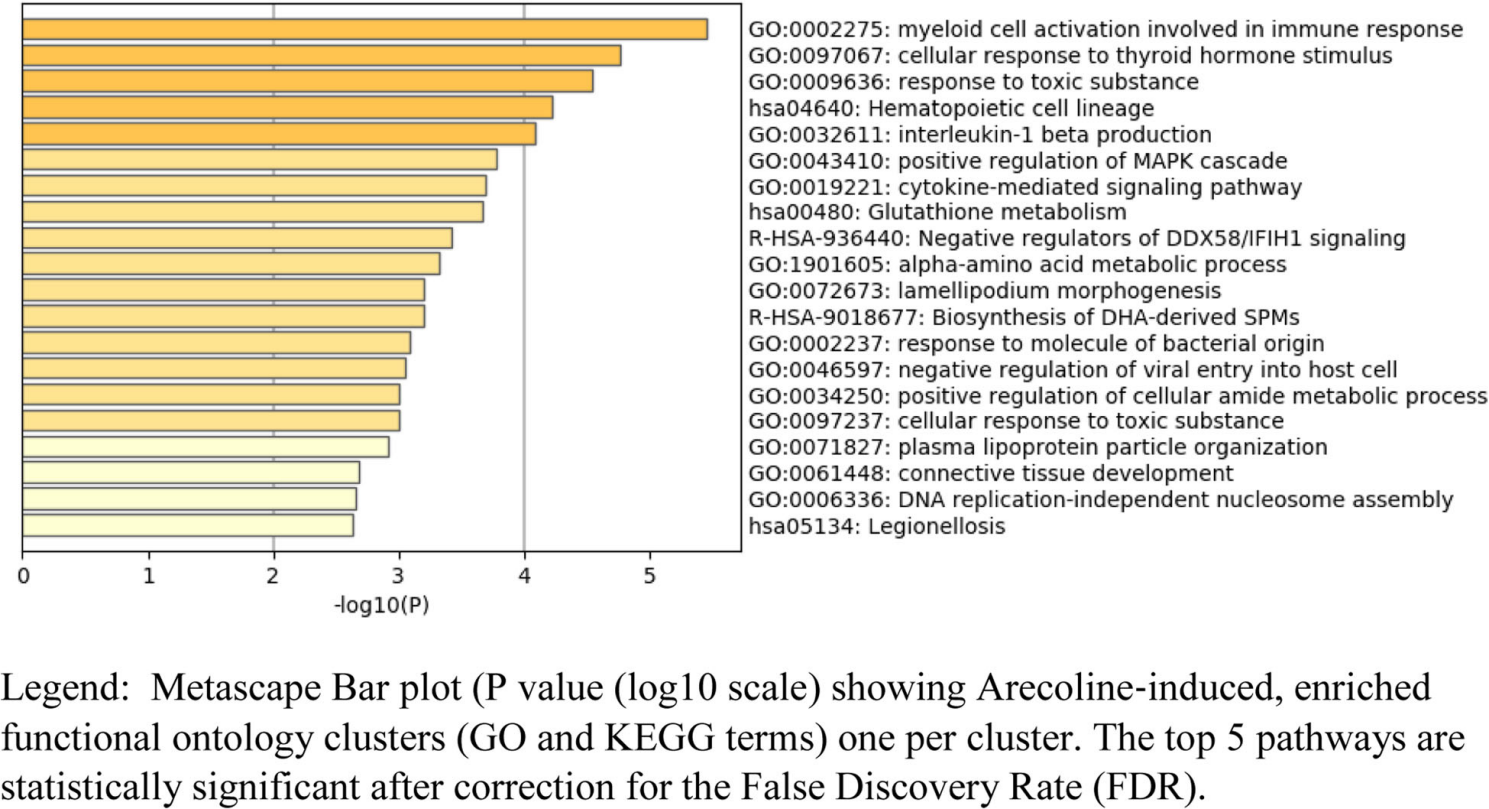
Pathway (Metascape) analysis following arecoline incubation

Pathway (Metascape) analysis of the 275 genes (Fig. 1) listed in the Additional Table 1, revealed 5 significant pathways after statistical correction by the false discovery rate (FDR): namely, myeloid cell activation involved in immune response, cellular response to thyroid hormone stimulus, responses to toxic substances and hematopoietic cell lineage.

#### Incubation with MNPA

359 gene hits were identified after incubation with MNPA with a *q* < 0.05 (Additional Table 2) reducing to 39 with a log-fold change of +/-1.5 (Table 2). The relevance of these genes to diabetes, obesity and/or metabolic syndrome was assessed independently by 2 of us (BJB and GAH) with reference to GeneCards, The Type 2 Diabetes Knowledge Portal and PubMed as described in the methods. Fourteen genes were identified of potential interest:

**Table 2:** Genes identified after incubation with MNPA

Gene Name	q-value^a^	logfc^b^	Gene Description
*H3F3AP4*	0.02	-6.71	H3 Histone Pseudogene 6
*PRKN*	0.05	-2.35	Parkin RBR E3 Ubiquitin Protein Ligase
*ARSEP1*	0.04	-2.28	Arylsulfatase L Pseudogene 1
*MYO7B*	0.03	-2.24	Myosin VIIB
*SIGLEC6*	0.04	-1.97	Sialic Acid Binding Ig Like Lectin 6
*WDR49*	0.01	-1.93	WD Repeat Domain 49
*TENM3*	0.04	-1.92	Teneurin Transmembrane Protein 3
*GLDN*	0.04	-1.90	Gliomedin
*GRIP1*	0.04	-1.78	Glutamate Receptor Interacting Protein 1
*NEGR1*	0.04	-1.73	Neuronal Growth Regulator 1
*LRMDA*	0.03	-1.72	Leucine Rich Melanocyte Differentiation Associated
*CCDC26*	0.05	-1.60	CCDC26 Long Non-Coding RNA
*AL023693.1*	0.05	-1.59	lncRNA
*KCNQ5*	0.04	-1.56	Potassium Voltage-Gated Channel Subfamily Q Member 5
*AL109914.1*	0.05	-1.54	lncRNA
*C2orf81*	0.05	-1.52	Chromosome 2 Open Reading Frame 81
*CNTN4*	0.02	-1.52	Contactin 4
*SLFN5*	0.03	1.54	Schlafen Family Member 5
*CRTAM*	0.04	1.55	Cytotoxic And Regulatory T-Cell Molecule
*NQO1*	0.02	1.59	NAD(P)H Quinone Dehydrogenase 1
*SEMA6B*	0.01	1.59	Semaphorin 6B
*INHBE*	0.03	1.60	Inhibin Subunit Beta E
*DLGAP1-AS2*	0.04	1.80	DLGAP1 Antisense RNA 2 lncRNA
*CLU*	0.03	1.90	Clusterin
*EFNB2*	0.04	1.90	Ephrin B2
*HTRA3*	0.04	1.90	HtrA Serine Peptidase 3
*SPTA1*	0.03	2.02	Spectrin Alpha, Erythrocytic 1
*HMOX1*	0.02	2.12	Heme Oxygenase 1
*LUCAT1*	0.03	2.13	Lung Cancer Associated Transcript 1
*OLAH*	0.03	2.17	Oleoyl-ACP Hydrolase
*TMEM140*	0.03	2.18	Transmembrane Protein 140
*NMRAL2P*	0.05	2.21	NmrA Like Redox Sensor 2, Pseudogene
*U62317.1*	0.04	2.33	Uncharacterized LOC105373098 RNA gene
*KLHDC7B*	0.04	2.41	Kelch Domain Containing 7B
*AL596330.1*	0.05	2.59	Subcategory (RNA class) for ENSG00000229400 Gene
*TREML3P*	0.01	2.68	Triggering Receptor Expressed On Myeloid Cells Like 3, Pseudogene
*SGCG*	0.03	2.76	Sarcoglycan Gamma
*NEUROD4*	0.03	2.78	Neuronal Differentiation 4
*TREML4*	0.03	2.88	Triggering Receptor Expressed On Myeloid Cells Like 4

Legend: Listed genes satisfied the following criteria *q* < 0.05^a^ and a log-fold change (logfc) of 1.5^b^

a Gliomedin (*GLDN* fold change 0.11)
b Glutamate Receptor Interacting Protein 1 (*GRIP1* fold change 0.15).
c Neuronal Growth Regulator 1 (*NEGR1* fold change 0.14).
d Potassium Voltage-Gated Channel Subfamily Q Member 5 (*KCNQ5* fold change 0.21).
e Cytotoxic And Regulatory T-Cell Molecule (*CRTAM* fold change 4.9).
f NAD(P)H Quinone Dehydrogenase 1 (*NQO1* fold change 4.9).
g Semaphorin 6B (*SEMA6B* fold change 4.9).
h Inhibin Subunit Beta E (*INHBE* fold change 5.4).
i Clusterin (*CLU* fold change 6.7).
j Spectrin Alpha, Erythrocytic 1 (*SPTA1* fold change 9.3).
k Heme Oxygenase 1 (*HMOX1* fold change 8.4).
l Transmembrane Protein 140 (*TMEM140* fold change 10.0).
m Sarcoglycan Gamma (*SGCG* fold change 24.8).
n Triggering Receptor Expressed On Myeloid Cells Like 4 (*TREML4* fold change 26.7).

**Fig. 2:**
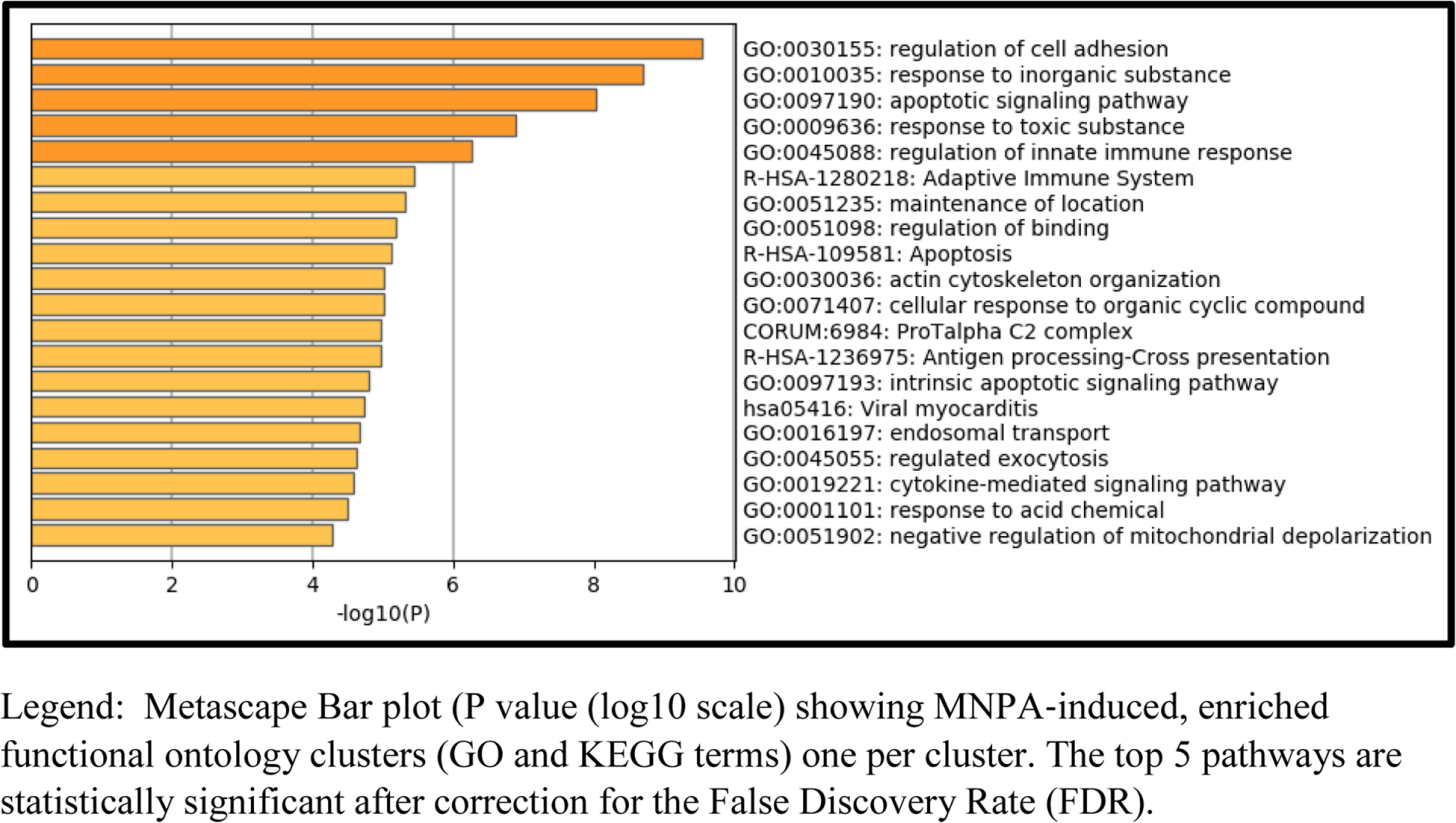
Pathway (Metascape) analysis following MNPA incubation

Pathway (Metascape) analysis of the 359 genes (Fig. 2) listed in the Additional Table 2, revealed 5 significant pathways after statistical correction by the FDR: namely, regulation of cell adhesion, response to inorganic substances, apoptotic signalling pathway, response to toxic substances and regulation of the innate immune response.

##### Gene expression changes in common with arecoline and MNPA

Using the strict criteria of *q* < 0.05 and log fold change of 1.5 there is only one overlapping gene, *H3F3AP4*, which is the H3 Histone Pseudogene 6 (chromosome location 2q31.1) of unknown function, although it might regulate gene function as a short interfering RNA or microRNA decoy with probable relevance to betel-induced carcinogenesis. Using the less stringent criteria of ‘*q* < 0.05 only’ then there are 69 of 275 (25 %) genes identified with arecoline that are also affected by its nitrosated product MNPA (Additional Fig. 4; data available from authors on request).

## Discussion

The aim of our study was to identify genes known to be associated with diabetes, obesity and metabolic syndrome whose expression was significantly altered by exposure to the arecal compounds arecoline and its nitrosated metabolite, MNPA. Whole transcriptome analysis by RNA-Seq of the human monocyte line THP1 did reveal a significant number of genes that are either downregulated or upregulated in response to incubation with arecoline or with MNPA. It was also hoped to determine whether the strength of the evidence for any of the genes found to be significantly affected might warrant further cell-based work and investigation amongst betel-chewing communities.

Consistent with the established effects of betel-quid chewing in humans, a number of cellular pathways and genes have been identified as being significantly affected by the arecal compounds used in our approach; these genes are known to relate to immune responses, to cell differentiation and lineage, to cellular responses to toxic and inorganic substances and to the development of obesity and T2D in humans. Other genes known to be associated with carcinogenesis were also found to have altered expression, as expected; some genes affected are associated with immune function and a smaller number of affected genes are related to neural development and could prove to be associated with susceptibility to addiction.

*Genes of specific relevance to obesity, diabetes and metabolic syndrome, based on published studies and genetic evidence, that showed good evidence suggesting that arecoline and/or MNPA may alter their expression, included * (Table 3): -.

**Table 3:** Genes with good or highly suggestive evidence of specific relevance to obesity, diabetes and metabolic syndrome

Gene	Disease Association or trait reported in humans	Putative mechanism
C-Type Lectin Domain Containing 10 A (*CLEC10A*)	Obesity, T2D	Adaptive and immune responses
Mitogen-Activated Protein Kinase 8 Interacting Protein 1 (*MAPK8IP1*)	T2D	Regulator of beta cell function and of immune function
Neuronal Growth Regulator 1 (*NEGR1*)	BMI, wait circumference, T2D	Cell adhesion
NAD(P)H Quinone Dehydrogenase 1 (*NQO1*)	Obesity, T2D. liver dysfunction	Anti-oxidant defences
Inhibin Subunit Beta E (*INHBE* )	Metabolic Syndrome, coronary heart disease	Cell proliferation, apoptosis, immune responses and hepatokine and hormone secretion
Glutamate receptor interacting protein 1 (*GRIP1*)	Metabolic syndrome (hepatic steatosis, hyperglycaemia and increased insulin resistance)	Macrophage and neuronal function and increased inflammation
Clusterin (*CLU*)	Glucose intolerance	Increased insulin resistance
Growth Factor Binding Protein 3 (*IGFBP3*)	Obesity, T2D, cerebrovascular disease	IGF binding protein affects vitamin D metabolism
Potassium Voltage-Gated Channel Subfamily Q Member 5 (*KCNQ5*)	Body mass index	Potassium channel
Cytotoxic And Regulatory T-Cell Molecule (*CRTAM*)	Body mass index and systolic hypertension	Innate immune system and determinant of insulin secretion
Spectrin (*SPTA1*)	HbA1c and T2D	Stability and structure of the cell membrane contributing to cell adhesion, cell spreading, and the cell cycle

Legend: *T2D* type 2 diabetes, *HbA1c* glycosylated haemoglobin

C-Type Lectin Domain Containing 10 A (*CLEC10A*), a calcium dependent endocytic receptor also known as the macrophage galactose-type lectin (MGL or CD301). It has been demonstrated to have a role in regulating adaptive and innate immune responses and is expressed in adipose tissue macrophages where it is associated with phenotypic switching of ATM subclasses in mice that then demonstrate either a lean or an obese phenotype [19]. Furthermore, evidence in humans demonstrates that missense and protein truncating mutations of the CLEC10A gene are strongly associated with the development of T2D (http://www.type2diabetesgenetics.org/gene/geneInfo/CLEC10A). In earlier rodent experiments MGL1 was found to be a novel regulator of monocyte trafficking in adipose tissue in response to dietary induced obesity [19, 20].

Mitogen-Activated Protein Kinase 8 Interacting Protein 1 (*MAPK8IP1*) gene encodes a regulator of pancreatic beta-cell function; it is also expressed in a large number of tissues including many associated with immune function and is a known trans-activator of the glucose transporter GLUT2. *MAPK8IP1* has a strong association with T2D with a missense mutation found in one family and, in *vitro*, that specific mutation has been found to be a key down-regulator of beta cell function [21].

Neuronal Growth Regulator 1 (*NEGR1*) is involved in cell adhesion. Multiple genome-wide association studies demonstrate strong genome-wide association (GWAS) signals for this gene with BMI, waist circumference and T2D [22] and certain mutations of this gene lead to Niemannn-Pick disease, a rare inherited metabolic disorder. (http://www.type2diabetesgenetics.org/gene/geneInfo/NEGR1). Furthermore, *NEGR1* knockout mice develop increased adiposity, including increased hepatocyte fat deposition, together with increases in glycaemia and in fasting serum insulin levels [23].

NAD(P)H Quinone Dehydrogenase 1 (*NQO1*) gene is a member of the NAD(P)H dehydrogenase (quinone) family, encodes a cytoplasmic 2-electron reductase (Entrez Gene) and is part of the antioxidant defence system. There is strong genetic evidence to support an association between *NQO1* variants by GWAS and increased risks of T2D and of an increased BMI (http://www.type2diabetesgenetics.org/gene/geneInfo/NQO1). NQO1 is highly expressed in human adipose tissue and its expression is reduced during diet-induced weight loss; furthermore its expression correlates directly with adiposity, glycaemia and markers of liver dysfunction [24]. Together, these findings indicate a role for NQO1 in the aetiology of obesity and T2D.

Inhibin Subunit Beta E (*INHBE*) gene is a member of the Transforming Growth Factor (TGF) beta superfamily. The transcribed peptide Activin E is ubiquitously expressed in a large number of normal tissues, many being known to be especially active during cell proliferation, apoptosis, immune response and hormone secretion. The highest expression is found in the liver where it acts as a hepatokine with effects on energy homeostasis in both brown and white adipose tissue [25]. The candidacy of the *INHBE* gene is further supported by strong GWAS signals associating it with cardiometabolic traits including raised serum triglycerides and overt coronary heart disease (http://www.type2diabetesgenetics.org/gene/geneInfo/INHBE).

*Other genes with evidence for relevance to T2D, Metabolic syndrome and obesity include the following (also included in* Table 3):

Glutamate receptor interacting protein 1 (*GRIP1*) mediates the trafficking and membrane organisation of a number of trans-membrane proteins in various cells including neurons and macrophages. Obese mice with a conditional knockout of *GRIP1* in macrophages develop massive macrophage infiltration and inflammation in many metabolically active tissues leading to many of the features that are associated with the metabolic syndrome such as hepatic steatosis, hyperglycaemia and increased insulin resistance [26]. Clusterin (*CLU*) is a molecular chaperone. Secretory clusterin is also known as ApoJ which has recently been identified as a novel hepatokine and deletion of hepatic ApoJ leads to insulin resistance and glucose tolerance [27]. Furthermore, in humans, serum ApoJ levels correlate directly with increases in insulin resistance but are decreased by rosiglitazone treatment [28]. Insulin Like Growth Factor Binding Protein 3 (*IGFBP3*) is the most abundant of 6 IGF-binding proteins. Important interactions have been observed between IGFBP3, vitamin D metabolism and obesity [29]. Furthermore, in people with T2D IGFB3 levels may inversely contribute to accelerated cerebrovascular disease [30]. Potassium Voltage-Gated Channel Subfamily Q Member 5 (*KCNQ5*) is a component of potassium channels and a strong GWAS association is seen between *KCNQ5* and body mass index (http://www.type2diabetesgenetics.org/gene/geneInfo/KCNQ5). Cytotoxic And Regulatory T-Cell Molecule (*CRTAM*) is a Protein Coding gene that has a role in the innate immune system and has also been proposed as a potential determinant of insulin secretion[31]. A strong GWAS association is seen between *CRTAM* and both body mass index and systolic blood pressure (http://www.type2diabetesgenetics.org/gene/geneInfo/CRTAM). Spectrin (*SPTA1*) is a component of the erythrocyte plasma membrane. A strong association is seen between *SPTA1* and separately with HbA1c (GWAS) and with T2D after adjustment for BMI (mainly missense mutations) (http://www.type2diabetesgenetics.org/gene/geneInfo/SPTA1).

A weakness of our approach is that the THP1 cell line is derived from a child with monocytic leukemia, however, in its favour is that these cells have been widely used to study monocyte and macrophage function [32]. Furthermore, their stable genetic background (compared to primary cells) with reproducible treatment responses makes this transformed cell line ideal for use in preliminary pilot studies aiming to justify further cellular and *in vivo* studies [33]. However, after incubation of THP1 cells with arecoline or MNPA we cannot, therefore, be sure if we are dealing with monocytes or macrophages as is described in the literature after PMA stimulation. Various compounds have been isolated and identified from *Areca catechu* nuts including alkaloids, tannins, flavones, triterpenes, steroids, and fatty acids [11]. We chose to focus on arecoline and two of the many nitrosated products of the arecal alkaloids (MNPA and MNPN), both because they are known to be the most carcinogenic of them and because low-dose nitrosamines cause T2D experimentally and in humans [12, 14]. Tissue and cell specific levels of arecoline derived from betel nut and its nitrosated products including MNPA are difficult to gauge. The average arecoline concentration in plasma in those chewing betel nut is 7.0 ng/ml (+/- SD of 10.7); in contrast the concentrations of MNPA are not reported [34]. The range of arecoline concentrations affecting different tissues adversely is clearly very wide as reviewed by Liu and colleagues [35] with a minimal active concentration/dose influencing macrophage expression *in vitro* of 10^− 5^ mol/l. Similarly, there is a wide range of concentrations (20–111 μg/ml) leading to minimal toxic effects on various cell types; 50 μg/ml had a minimal toxic effect on mice bone marrow and human peripheral blood lymphocytes generating reactive oxygen species [36]. Hence the administered concentrations in our experiments are consistent with the literature, but nonetheless, we cannot be certain that the concentrations we used are definitely physiologically relevant, which is a limitation of our approach.

Unfortunately, for technical reasons, we did not get results with MNPN. *Areca catechu* chewing quids often contain various other additives such as slaked lime, spices, sweeteners, and are wrapped in leaves of the *Piper betle* vine; furthermore, in many countries other than Taiwan they often contain chewing tobacco [37]. We cannot therefore exclude the possibility that many major effects of chewing betel quids in humans may be due to ingestion of betel quid components other than those from the *Areca catechu* nut. However, obesity and hyperglycaemia were induced in CD1 mice fed *Areca catechu* nut without any other betel quid component [9] and this data contributed to our focus on the findings for genes associated with those particular disorders in humans.

In the biostatistical analysis we have been conservative in applying a *q* value of < 0.05 and a log fold change of x1.5 in order to maximise the chances of identifying relevant transcripts from our exploratory genome-wide experiments. Further experiments will be required to assess the effect of either upregulation or downregulation of these genes in suitable models for detecting changes in function of the particular genes identified in this way.

In summary, the gene expression profiles described, induced by arecoline and MNPA derived from *Areca catechu*-(Betel-nut) would act through a number of mechanisms including regulation of adaptive and immune responses, transactivation of GLUT2, anti-oxidant defences and secretion of hepatocytokines. We would, therefore, hypothesise that the cumulative effect of betel-nut ingestion in humans, through the described changes in gene expression, would contributes to a predisposition to obesity, T2D and thereby, hepatic steatosis and thereby to an increased risk of cardiovascular disease. This would be supported by the population and rodent studies as noted in the introduction.

## Conclusions

This pilot study has identified a large number of genes whose expression was changed significantly in human TPH1 cells following incubation with arecoline and MNPA and that are known to be associated with increased risks of obesity and T2D in humans. These findings suggest that further investigation of these genes is warranted both *in vitro* and in humans from betel-chewing communities, and that our findings should be placed in the public domain so that they are available to the scientific community.

## Methods

The aim of our pilot study was to identify gene expression relevant to obesity, T2D and the metabolic syndrome using a genome-wide transcriptomic approach in a human monocyte cell line incubated with arecoline and its nitrosated products.

The THP1 (human acute monocytic leukemia derived; ATCC number TIB-202 purchased from Thermofisher) cell line [38] was regularly maintained in RPMI 1640 medium containing GlutaMAX™, supplemented with 10 % FCS (Gibco™ Newborn Calf Serum [heat inactivated], of New Zealand origin; Thermo Fisher Scientific), 5 % AA (Gibco® MEM), Non-Essential Amino Acids, 100 U/mL penicillin and 100 µg/mL streptomycin (Thermo Fisher Scientific). Cells were grown at 37 °C in a humidified atmosphere of 5 % CO2 in air, and sub-passaged with fresh complete RPMI medium every three days. The cell line was regularly checked to be mycoplasma free using the VenorGeM Mycoplasma detection kit (Cambio Ltd, UK) according to manufacturer’s instructions.

Stock solutions of Arecoline (100 mg/ml of methanol), MNPA (2.5 mg/ml of methanol), MNPN (20 mg/ml of 100 % ethyl acetate) and PMA((Phorbol-12-Myrsitate-13-Acetate (2 mg/ml of DMSO)) were made, aliquoted and stored at -20^o^C. To assess the toxicity of these preparations, THP-1 cells was incubated with 10-fold serial dilutions of arecoline, MNPN as well as MNPA in culture media. The cells were then observed under phase contrast light microscopy for up to 6 days. We then selected the highest non-toxic concentration listed below for our experiments. 1 × 10^6^ THP1 cells were treated with either 50 μg/ml Arecoline, 25 μg/ml MNPA, 200 μg/ml MNPN or 200 ng/ml PMA as a positive control in 6-well plates and cells were harvested after 6 h, 24 h and 48 h of treatment. Methanol and ethyl acetate were used as negative controls. Three independent experiments were performed for each exposure. Cell viability and proliferation was assessed by phase contrast light microscopy and using a MTT assay. All chemicals were purchased from Sigma-Aldrich.

### RNA extraction and RT-qPCR for gene expression of human TNFa, IL-6 and IL-8 analysis

Prior to whole genome sequencing we sought to confirm the biological viability of the THP1 cells studied following their incubation with arecoline, MNPA or MNPN by measuring their cytokine responses. Total RNA was extracted from treated cells using QIAGEN RNeasy Kit according to manufacturer’s instructions. cDNA was synthesized using 1 µg of the extracted RNA with an Oligo (dT) primer using a SuperScript® IV First-Strand Synthesis System (Thermo Fisher Scientific) as follows: primer annealing at 65 °C for 5 min; RNA reverse transcriptase at 50 °C for 1 h 10 min and at 70 °C for 15 min. The cDNA was used as a template to determine the expression level of human TNFa, IL-6, IL-8 and 18 S [39] treated (arecoline, MNPA or MNPN) /untreated THP1 cells. The RT-PCR was performed on StepOne™ Real-Time PCR System thermal cycler (Applied Biosystems™). Each PCR reaction consisted of 2 µl of cDNA, 2X SYBR® Green JumpStart™ Taq ReadyMix™ (Sigma-Aldrich), 0.2 µM of each forward and reverse primers (Additional Table 3). qPCR reaction conditions were: cDNA denaturation at 95 °C for 5 min, cDNA amplification at 95 °C for 15 s, primer annealing at 62 °C for 1 min for 45 cycles, then melt curves were obtained at 95 °C for 15 s, 60 °C for 1 min and a final step at 95 °C for 15 s. All target genes were normalised to *18 S RNA* using the standard *ΔΔ*Ct method. Results were analysed with Thermo Fisher StepOne software v2.3. Each experiment was performed in triplicate and fold change expression level was reported relative to 18 S level.

### RNA-sequencing and bioinformatics

Fragmented cDNA Sequencing libraries were generated from 100ng of Total RNA using NEBNext Ultra II with polyA isolation module (Illumina, San Diego, California, USA) according to manufacturer’s protocol. cDNA quantity and quality were evaluated using the Qubit dsDNA HS assay kit (Thermo Fisher Scientific, Erembodegem-Aalst, Belgium). Size distribution of our library was determined using an Agilent 2100 Bioanalyser. Pooled indexed paired-end libraries were sequenced on the Illumina NextSeq 500 (Illumina, USA) using the manufacturer’s instructions. Sequencing was performed at Queen Mary University of London Genome Centre core facility in the Blizard Institute London.

Sequenced reads were mapped using Kallisto [40] with default settings. Mean insert sizes and standard deviations were provided as input. Analysis of differential gene expression was performed using sleuth, applying a generalized linear model and utilising bootstraps on reads to estimate inferential variance. Genome-wide corrected p-values were calculated using the Bonferroni multiple testing adjustment procedure.

Functional annotation as well as pathway enrichment analyses were performed using DAVID, Reactome and Metascape (https://metascape.org/gp/index.html#/main/step1).

Candidacy of genes identified were assessed by look-ups in:

1. GeneCards: The human genome database (https://www.genecards.org/) to check for alias’s, gene summary (Entrez, Genecards and UniProtKB/Swiss-Prot) and mRNA expression in normal human tissues (GTEx, Illumina, BioGPS).
2. Type 2 diabetes knowledge portal (http://www.type2diabetesgenetics.org/). Genes considered were only those with ‘strong’ evidence for signal defined by either at least one variant within the coding sequence ± 100 kb that is associated with at least one phenotype with a *p* value < 5e-8 identified by a genome-wide association scan (GWAS), or at least one known variant with a missense or protein truncating mutation in the encoded protein that is associated with at least one phenotype with a *p* value < 5e-6.
3. PubMed (GAH and BJB independently) by searching biology of the identified gene and biological relevance to obesity, T2D or the metabolic syndrome before collation of results.

## Supplementary Information

**Additional file 1: Figure 1.** Phase light microscopy of THP-1 cells. Individual panels are of control cells or various concentration of arecoline studied at 48 h.

**Additional file 2: Figure 2. **MTT-Cell proliferation assay. Bar chart with 95% confidence limits plotting % cell viability by control and after incubation of arecoline/well at varying concentrations.

**Additional file 3: Figure 3. **Betel nut compounds treatment in THP1 cells. Bar chart with 95% confidence limits plotting a time course for TNFa, IL6 and IL8 mRNA (normalised relative to 18S RNA) in response to incubation with arecoline, MNPA, MNPN and PMA.

**Additional file 4: Figure 4.** Shared genes expressed (*q* < 0.05) after incubation with either arecoline or MNPA. Illustrated as a Venn diagram.

**Additional file 5: Table 1. **All genes identified after incubation with arecoline with significant increased expression after Bonferroni correction. **Table 2.** Genes identified after incubation with MNPA with significant increased expression after Bonferroni correction. **Table S3.** Primer sequences used in qPCR analysis https://www.ncbi.nlm.nih.gov/pmc/articles/PMC2851245/

## Data Availability

All genes identified after incubation with significant expression changes after Bonferroni correction, irrespective of candidacy, are listed in Additional Tables 2 and 3. The full dataset generated and/or analysed during the current study are available in the GEO repository with an accession number of GSE179143, website: https://www.ncbi.nlm.nih.gov/geo/query/acc.cgi?acc=GSE179143.

## Abbreviations

MNPA: 3-methylnitrosaminopropionaldehyde
MNPN: 3-methylnitrosaminopropionitrile
PMA: Phorbol-12-Myrsitate-13-Acetate
T2D: Type 2 diabetes
T1D: Type 1 diabetes
RR: Relative risk
