# Supplementary material for: Areca catechu-(Betel-nut)-induced whole transcriptome changes in a human monocyte cell line that may have relevance to diabetes and obesity; a pilot study
Source: BMC Endocr Disord. 2021 Aug 14;21:165. doi: 10.1186/s12902-021-00827-1 (PMC8364090; doi:10.1186/s12902-021-00827-1)
Supplement: Supplementary file 1 — Additional file 1: Figure 1. Phase light microscopy of THP-1 cells. Individual panels are of control cells or various concentration of arecoline studied at 48 h. [file 12902_2021_827_MOESM1_ESM.pptx]

## Slide 1
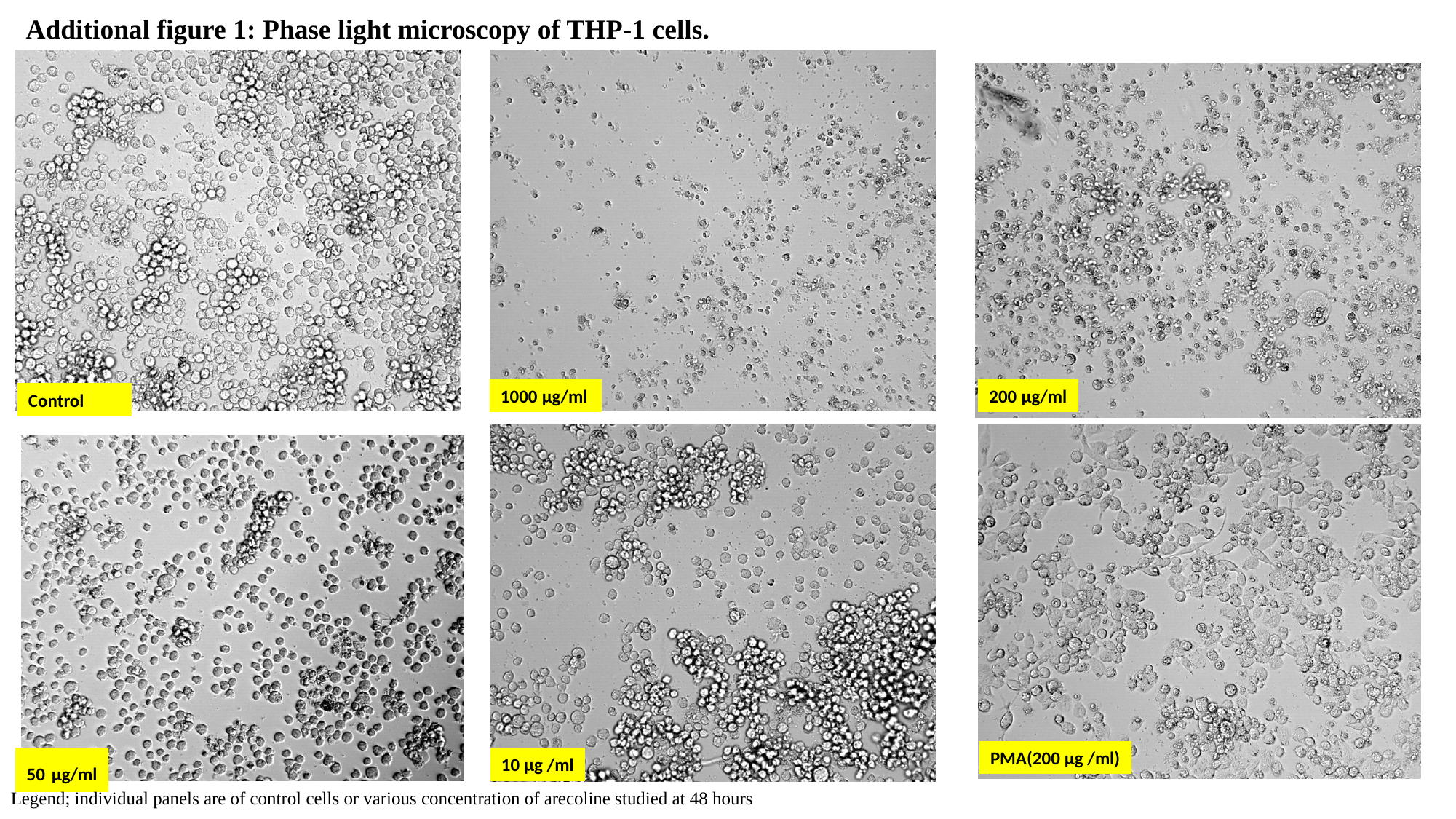

# Additional figure 1: Phase light microscopy of THP-1 cells.
1000 µg/ml
200 µg/ml
Control
PMA(200 µg /ml)
10 µg /ml
50 µg/ml
Legend; individual panels are of control cells or various concentration of arecoline studied at 48 hours
