# Supplementary figures and images for: Areca catechu-(Betel-nut)-induced whole transcriptome changes in a human monocyte cell line that may have relevance to diabetes and obesity; a pilot study
Source: BMC Endocr Disord. 2021 Aug 14;21:165. doi: 10.1186/s12902-021-00827-1 (PMC8364090; doi:10.1186/s12902-021-00827-1)

## Slide 1
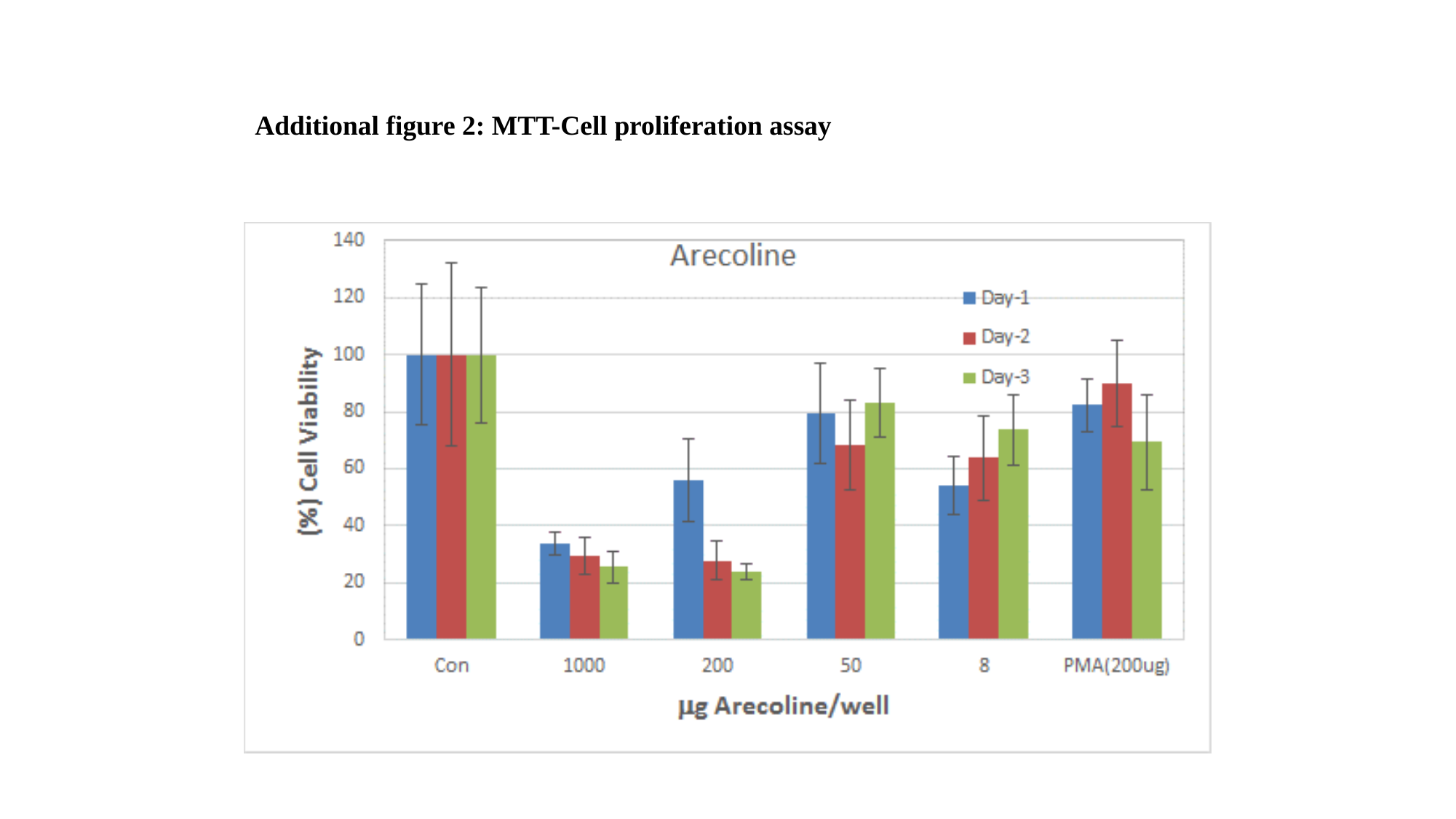

# Additional figure 2: MTT-Cell proliferation assay

Supplement: Supplementary file 2 — Additional file 2: Figure 2. MTT-Cell proliferation assay. Bar chart with 95% confidence limits plotting % cell viability by control and after incubation of arecoline/well at varying concentrations. [file 12902_2021_827_MOESM2_ESM.pptx]
