# Supplementary material for: Areca catechu-(Betel-nut)-induced whole transcriptome changes in a human monocyte cell line that may have relevance to diabetes and obesity; a pilot study
Source: BMC Endocr Disord. 2021 Aug 14;21:165. doi: 10.1186/s12902-021-00827-1 (PMC8364090; doi:10.1186/s12902-021-00827-1)
Supplement: Supplementary file 3 — Additional file 3: Figure 3. Betel nut compounds treatment in THP1 cells. Bar chart with 95% confidence limits plotting a time course for TNFa, IL6 and IL8 mRNA (normalised relative to 18S RNA) in response to incubation with arecoline, MNPA, MNPN and PMA. [file 12902_2021_827_MOESM3_ESM.pptx]

## Slide 1
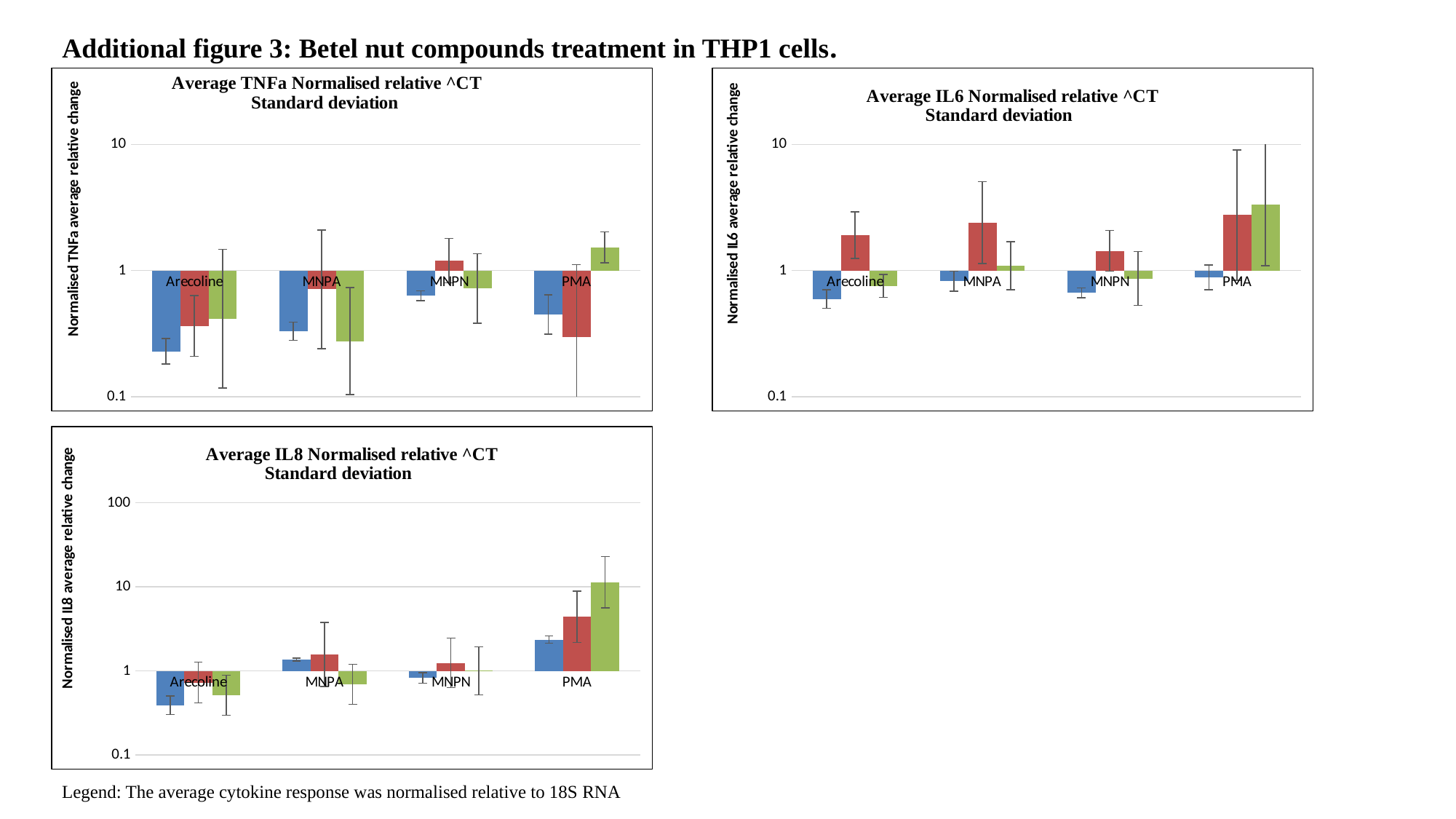

# Additional figure 3: Betel nut compounds treatment in THP1 cells.
### Chart: Average TNFa Normalised relative ^CT
Standard deviation
| Category | 6hrs | 24hrs | 48hrs |
|---|---|---|---|
| Arecoline | 0.22894284851066637 | 0.3634241185664279 | 0.41601676461038084 |
| MNPA | 0.33019272488946266 | 0.7113786608980125 | 0.27589241763811206 |
| MNPN | 0.6316359597656379 | 1.2 | 0.7211247851537042 |
| PMA | 0.44814047465571644 | 0.29624960684073703 | 1.5274930108978468 |
### Chart: Average IL6 Normalised relative ^CT
Standard deviation
| Category | 6hrs | 24hrs | 48hrs |
|---|---|---|---|
| Arecoline | 0.5943921952763129 | 1.9078163691030556 | 0.7559526299369239 |
| MNPA | 0.8242570599617113 | 2.4096831019876706 | 1.090272355699284 |
| MNPN | 0.6649399761150976 | 1.4325697513313385 | 0.8662391053409028 |
| PMA | 0.8849344009597906 | 2.7616803462223563 | 3.3330021893264057 |
### Chart: Average IL8 Normalised relative ^CT
Standard deviation
| Category | 6hrs | 24hrs | 48hrs |
|---|---|---|---|
| Arecoline | 0.3914867641168864 | 0.7268482371328558 | 0.5129927840030091 |
| MNPA | 1.3658399388865803 | 1.570014469689623 | 0.6952053289772899 |
| MNPN | 0.8276772529143362 | 1.2454769869555096 | 1.002659586992917 |
| PMA | 2.3588469901582667 | 4.4060522772299775 | 11.379371744705272 |Legend: The average cytokine response was normalised relative to 18S RNA
