# Supplementary material for: Areca catechu-(Betel-nut)-induced whole transcriptome changes in a human monocyte cell line that may have relevance to diabetes and obesity; a pilot study
Source: BMC Endocr Disord. 2021 Aug 14;21:165. doi: 10.1186/s12902-021-00827-1 (PMC8364090; doi:10.1186/s12902-021-00827-1)
Supplement: Supplementary file 4 — Additional file 4: Figure 4. Shared genes expressed (q < 0.05) after incubation with either arecoline or MNPA. Illustrated as a Venn diagram. [file 12902_2021_827_MOESM4_ESM.pptx]

## Slide 1
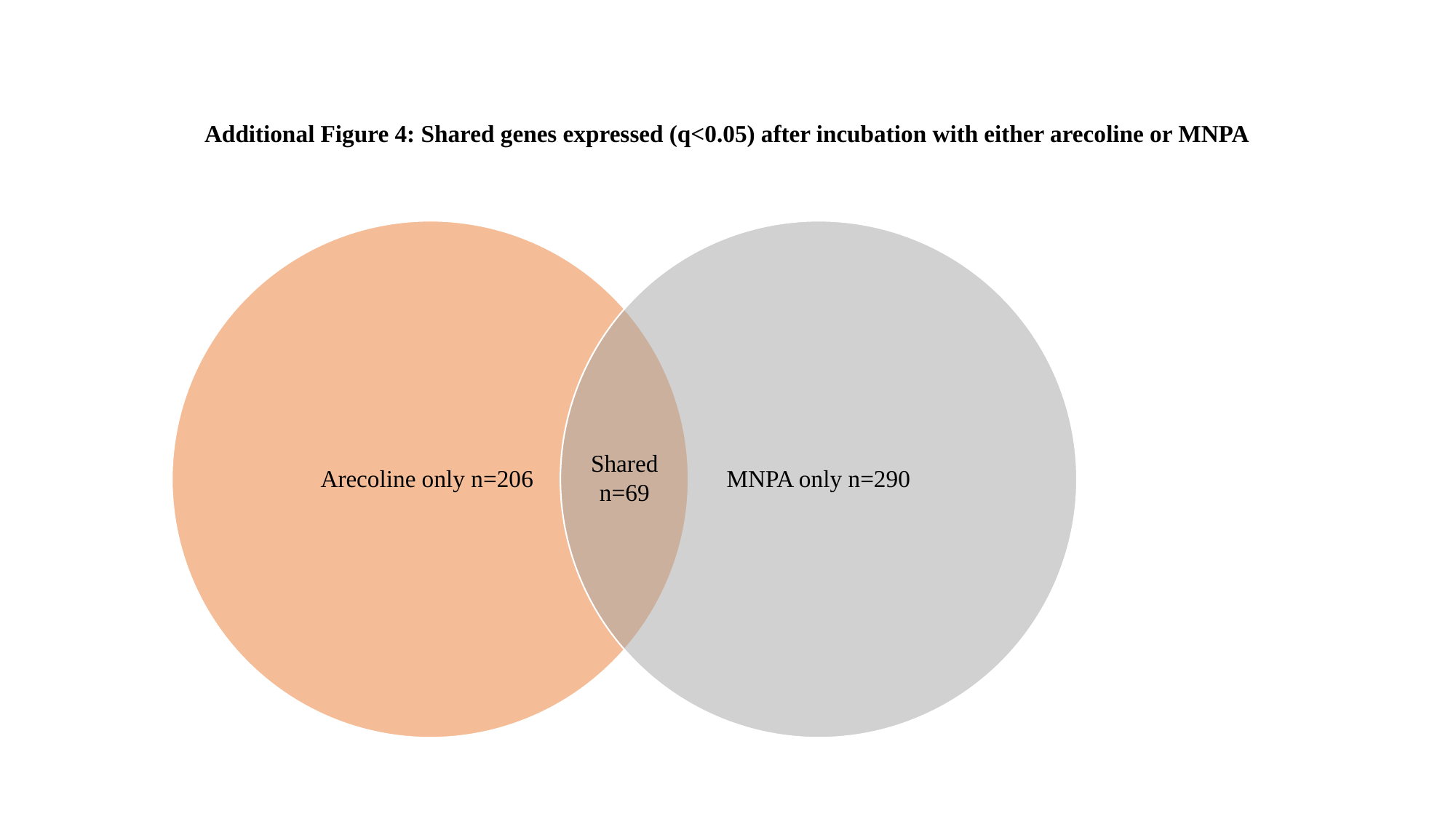

# Additional Figure 4: Shared genes expressed (q<0.05) after incubation with either arecoline or MNPA
Shared
n=69
