# Supplementary material for: Areca catechu-(Betel-nut)-induced whole transcriptome changes in a human monocyte cell line that may have relevance to diabetes and obesity; a pilot study
Source: BMC Endocr Disord. 2021 Aug 14;21:165. doi: 10.1186/s12902-021-00827-1 (PMC8364090; doi:10.1186/s12902-021-00827-1)
Supplement: Supplementary file 5 — Additional file 5: Table 1. All genes identified after incubation with arecoline with significant increased expression after Bonferroni correction. Table 2. Genes identified after incubation with MNPA with significant increased expression after Bonferroni correction. Table S3. Primer sequences used in qPCR analysis https://www.ncbi.nlm.nih.gov/pmc/articles/PMC2851245/ [file 12902_2021_827_MOESM5_ESM.docx]

**Additional table 1**

**All genes identified after incubation with arecoline with significant increased expression after Bonferroni correction**

| \| Gene Name \| Gene ID \| \| --- \| --- \| \| H3F3AP4 \| ENSG00000235655 \| \| MEP1A \| ENSG00000112818 \| \| KBTBD11-OT1 \| ENSG00000283239 \| \| IGFBP3 \| ENSG00000146674 \| \| AC097372.1 \| ENSG00000250673 \| \| PTCRA \| ENSG00000171611 \| \| IL3RA \| ENSG00000185291 \| \| CLEC10A \| ENSG00000132514 \| \| ACKR3 \| ENSG00000144476 \| \| AC113133.1 \| ENSG00000253389 \| \| JUP \| ENSG00000173801 \| \| CCDC188 \| ENSG00000234409 \| \| MAPK8IP1 \| ENSG00000121653 \| \| MATK \| ENSG00000007264 \| \| ADRB2 \| ENSG00000169252 \| \| AC020909.2 \| ENSG00000268518 \| \| GDF11 \| ENSG00000135414 \| \| FAM27C \| ENSG00000154537 \| \| IL4I1 \| ENSG00000104951 \| \| KIT \| ENSG00000157404 \| \| AC100858.3 \| ENSG00000255491 \| \| CSPG4 \| ENSG00000173546 \| \| LTC4S \| ENSG00000213316 \| \| KRT17P1 \| ENSG00000131885 \| \| TNFAIP3 \| ENSG00000118503 \| \| LINC01632 \| ENSG00000277199 \| \| ZNF702P \| ENSG00000242779 \| \| C1orf127 \| ENSG00000175262 \| \| LIPG \| ENSG00000101670 \| \| PDPN \| ENSG00000162493 \| \| LINC00482 \| ENSG00000185168 \| \| KRT17P2 \| ENSG00000186831 \| \| TRAF5 \| ENSG00000082512 \| \| PTX3 \| ENSG00000163661 \| \| PLA2G4C \| ENSG00000105499 \| \| ANXA8 \| ENSG00000265190 \| \| TMEM80 \| ENSG00000177042 \| \| LINC00680 \| ENSG00000215190 \| \| SCAMP1-AS1 \| ENSG00000245556 \| \| TMEM231 \| ENSG00000205084 \| \| C20orf197 \| ENSG00000176659 \| \| SRRM2-AS1 \| ENSG00000205913 \| \| AFF2 \| ENSG00000155966 \| \| TMEM147-AS1 \| ENSG00000236144 \| \| ALOX5 \| ENSG00000012779 \| \| NPTXR \| ENSG00000221890 \| \| WIPF3 \| ENSG00000122574 \| \| AC090796.1 \| ENSG00000253821 \| \| RGS16 \| ENSG00000143333 \| \| SMPD2 \| ENSG00000135587 \| \| ITGB2-AS1 \| ENSG00000227039 \| \| COL27A1 \| ENSG00000196739 \| \| AC139491.2 \| ENSG00000248596 \| \| ISG15 \| ENSG00000187608 \| \| STAG3 \| ENSG00000066923 \| \| TRIM22 \| ENSG00000132274 \| \| DANT2 \| ENSG00000235244 \| \| TTC3P1 \| ENSG00000215105 \| \| SLAMF8 \| ENSG00000158714 \| \| DHRS12 \| ENSG00000102796 \| \| PTGIR \| ENSG00000160013 \| \| EPHX2 \| ENSG00000120915 \| \| PCDH9 \| ENSG00000184226 \| \| AC073957.3 \| ENSG00000273151 \| \| VILL \| ENSG00000136059 \| \| RGMA \| ENSG00000182175 \| \| ANGPTL6 \| ENSG00000130812 \| \| MCRIP1 \| ENSG00000225663 \| \| MAF \| ENSG00000178573 \| \| AC060780.1 \| ENSG00000267002 \| \| FRAT1 \| ENSG00000165879 \| \| ANXA2R \| ENSG00000177721 \| \| TBL3 \| ENSG00000183751 \| \| IQCD \| ENSG00000166578 \| \| C1RL \| ENSG00000139178 \| \| NUDT16P1 \| ENSG00000246082 \| \| IFIH1 \| ENSG00000115267 \| \| DUXAP10 \| ENSG00000244306 \| \| HIST2H2BF \| ENSG00000203814 \| \| PDGFD \| ENSG00000170962 \| \| RABL2B \| ENSG00000079974 \| \| CXCL1 \| ENSG00000163739 \| \| SIRPB2 \| ENSG00000196209 \| \| TP53I3 \| ENSG00000115129 \| \| SMIM4 \| ENSG00000168273 \| \| SLC27A3 \| ENSG00000143554 \| \| NHSL2 \| ENSG00000204131 \| \| CES3 \| ENSG00000172828 \| \| BAG1 \| ENSG00000107262 \| \| MS4A7 \| ENSG00000166927 \| \| CD44 \| ENSG00000026508 \| \| AC093668.2 \| ENSG00000272949 \| \| ARRB1 \| ENSG00000137486 \| \| ATP5S \| ENSG00000125375 \| \| ZNF655 \| ENSG00000197343 \| \| CD209 \| ENSG00000090659 \| \| THBS3 \| ENSG00000169231 \| \| DGAT1 \| ENSG00000185000 \| \| CYB561A3 \| ENSG00000162144 \| | Gene Name Gene ID   \| CHI3L1 \| ENSG00000133048 \| \| --- \| --- \| \| MZF1 \| ENSG00000099326 \| \| GATA2-AS1 \| ENSG00000244300 \| \| ARHGEF9 \| ENSG00000131089 \| \| UBA7 \| ENSG00000182179 \| \| LENG1 \| ENSG00000105617 \| \| NFKB2 \| ENSG00000077150 \| \| AC078846.1 \| ENSG00000273329 \| \| HIST2H4A \| ENSG00000270882 \| \| CACNA1A \| ENSG00000141837 \| \| MTHFD1L \| ENSG00000120254 \| \| GALT \| ENSG00000213930 \| \| DUXAP8 \| ENSG00000206195 \| \| ASGR2 \| ENSG00000161944 \| \| KCNMB1 \| ENSG00000145936 \| \| AL603839.3 \| ENSG00000238287 \| \| RWDD2B \| ENSG00000156253 \| \| ASS1 \| ENSG00000130707 \| \| GSDMD \| ENSG00000104518 \| \| GSN \| ENSG00000148180 \| \| C21orf2 \| ENSG00000160226 \| \| DENND6B \| ENSG00000205593 \| \| ARMCX1 \| ENSG00000126947 \| \| IFITM3 \| ENSG00000142089 \| \| TREM2 \| ENSG00000095970 \| \| THSD7A \| ENSG00000005108 \| \| FSCN1 \| ENSG00000075618 \| \| SURF1 \| ENSG00000148290 \| \| RIN1 \| ENSG00000174791 \| \| PCED1A \| ENSG00000132635 \| \| ZNF521 \| ENSG00000198795 \| \| ABHD14B \| ENSG00000114779 \| \| PLD4 \| ENSG00000166428 \| \| SPNS3 \| ENSG00000182557 \| \| GALNT12 \| ENSG00000119514 \| \| CATSPER1 \| ENSG00000175294 \| \| PIGO \| ENSG00000165282 \| \| RRP12 \| ENSG00000052749 \| \| ZDHHC8 \| ENSG00000099904 \| \| HLA-DMA \| ENSG00000204257 \| \| ACCS \| ENSG00000110455 \| \| OPTN \| ENSG00000123240 \| \| CKB \| ENSG00000166165 \| \| PYCR1 \| ENSG00000183010 \| \| PARVG \| ENSG00000138964 \| \| SATB1 \| ENSG00000182568 \| \| QTRT1 \| ENSG00000213339 \| \| YBX3 \| ENSG00000060138 \| \| IFI35 \| ENSG00000068079 \| \| AL035446.1 \| ENSG00000234147 \| \| STAG3L3 \| ENSG00000174353 \| \| CYTH4 \| ENSG00000100055 \| \| PLA2G7 \| ENSG00000146070 \| \| CX3CR1 \| ENSG00000168329 \| \| AC215219.1 \| ENSG00000226210 \| \| GRK3 \| ENSG00000100077 \| \| TSTA3 \| ENSG00000104522 \| \| ANPEP \| ENSG00000166825 \| \| IMPDH2 \| ENSG00000178035 \| \| SNHG15 \| ENSG00000232956 \| \| TRPM4 \| ENSG00000130529 \| \| PHYKPL \| ENSG00000175309 \| \| MMAB \| ENSG00000139428 \| \| AC005154.1 \| ENSG00000196295 \| \| GMEB1 \| ENSG00000162419 \| \| SHMT2 \| ENSG00000182199 \| \| SH3BP2 \| ENSG00000087266 \| \| WASF1 \| ENSG00000112290 \| \| Sep-06 \| ENSG00000125354 \| \| DISC1 \| ENSG00000162946 \| \| ALDH2 \| ENSG00000111275 \| \| TSPYL4 \| ENSG00000187189 \| \| PSMB8-AS1 \| ENSG00000204261 \| \| ERMAP \| ENSG00000164010 \| \| STX10 \| ENSG00000104915 \| \| ALAS1 \| ENSG00000023330 \| \| METTL3 \| ENSG00000165819 \| \| MRPL54 \| ENSG00000183617 \| \| CTSH \| ENSG00000103811 \| \| FLT3 \| ENSG00000122025 \| \| C12orf57 \| ENSG00000111678 \| \| PSMB9 \| ENSG00000240065 \| \| SHOX2 \| ENSG00000168779 \| \| PDXK \| ENSG00000160209 \| \| PPP2R5D \| ENSG00000112640 \| \| TNKS2 \| ENSG00000107854 \| \| GINM1 \| ENSG00000055211 \| \| UBE2A \| ENSG00000077721 \| \| WSB1 \| ENSG00000109046 \| \| MEGF9 \| ENSG00000106780 \| \| ZHX1 \| ENSG00000165156 \| \| ZBTB2 \| ENSG00000181472 \| \| RPA2 \| ENSG00000117748 \| \| SNRPA1 \| ENSG00000131876 \| \| WDR82 \| ENSG00000164091 \| \| HSPA14 \| ENSG00000187522 \| \| COL4A3BP \| ENSG00000113163 \| \| ADAM9 \| ENSG00000168615 \| \| TOMM34 \| ENSG00000025772 \| | Gene Name Gene ID   \| HERC4 \| \| ENSG00000148634 \| \| \| --- \| --- \| --- \| --- \| \| ANXA7 \| \| ENSG00000138279 \| \| \| MPV17L2 \| \| ENSG00000254858 \| \| \| DCAF6 \| \| ENSG00000143164 \| \| \| RCN2 \| \| ENSG00000117906 \| \| \| KIF11 \| \| ENSG00000138160 \| \| \| CREG1 \| \| ENSG00000143162 \| \| \| GTF2A1 \| \| ENSG00000165417 \| \| \| C20orf24 \| \| ENSG00000101084 \| \| \| CTSL \| \| ENSG00000135047 \| \| \| INIP \| \| ENSG00000148153 \| \| \| ARRDC4 \| \| ENSG00000140450 \| \| \| CD55 \| \| ENSG00000196352 \| \| \| DCLRE1A \| \| ENSG00000198924 \| \| \| PGRMC1 \| \| ENSG00000101856 \| \| \| NINJ1 \| \| ENSG00000131669 \| \| \| SLF2 \| \| ENSG00000119906 \| \| \| SLC5A3 \| \| ENSG00000198743 \| \| \| UHMK1 \| \| ENSG00000152332 \| \| \| UPF3B \| \| ENSG00000125351 \| \| \| ORC6 \| \| ENSG00000091651 \| \| \| HLTF \| \| ENSG00000071794 \| \| \| C1orf162 \| \| ENSG00000143110 \| \| \| CEP55 \| \| ENSG00000138180 \| \| \| SLC30A1 \| \| ENSG00000170385 \| \| \| PDRG1 \| \| ENSG00000088356 \| \| \| PAAF1 \| \| ENSG00000175575 \| \| \| CENPU \| \| ENSG00000151725 \| \| \| C1orf198 \| \| ENSG00000119280 \| \| \| OIP5 \| \| ENSG00000104147 \| \| \| FLNB \| \| ENSG00000136068 \| \| \| ZDHHC14 \| \| ENSG00000175048 \| \| \| ASF1A \| \| ENSG00000111875 \| \| \| SLA \| \| ENSG00000155926 \| \| \| HBS1L \| \| ENSG00000112339 \| \| \| LRRC42 \| \| ENSG00000116212 \| \| \| WDHD1 \| \| ENSG00000198554 \| \| \| CREM \| \| ENSG00000095794 \| \| \| HSPH1 \| \| ENSG00000120694 \| \| \| ABHD3 \| \| ENSG00000158201 \| \| \| MAP3K20 \| \| ENSG00000091436 \| \| \| CASP3 \| \| ENSG00000164305 \| \| \| AIDA \| \| ENSG00000186063 \| \| \| TNFAIP2 \| \| ENSG00000185215 \| \| \| MSC-AS1 \| \| ENSG00000235531 \| \| \| FZD5 \| \| ENSG00000163251 \| \| \| AC092718.4 \| \| ENSG00000261061 \| \| \| ME1 \| \| ENSG00000065833 \| \| \| G6PD \| \| ENSG00000160211 \| \| \| RAB23 \| \| ENSG00000112210 \| \| \| C9orf72 \| \| ENSG00000147894 \| \| \| PGD \| \| ENSG00000142657 \| \| \| NEIL3 \| \| ENSG00000109674 \| \| \| EIF1B \| \| ENSG00000114784 \| \| \| RIT1 \| \| ENSG00000143622 \| \| \| L2HGDH \| \| ENSG00000087299 \| \| \| GSR \| \| ENSG00000104687 \| \| \| TXNRD1 \| \| ENSG00000198431 \| \| \| LILRA5 \| \| ENSG00000187116 \| \| \| GCLM \| \| ENSG00000023909 \| \| \| TMTC3 \| \| ENSG00000139324 \| \| \| NMT2 \| \| ENSG00000152465 \| \| \| SUCNR1 \| \| ENSG00000198829 \| \| \| SRXN1 \| \| ENSG00000271303 \| \| \| SULT4A1 \| \| ENSG00000130540 \| \| \| B4GALNT1 \| \| ENSG00000135454 \| \| \| NQO1 \| \| ENSG00000181019 \| \| \| PIR \| \| ENSG00000087842 \| \| \| TMEM191A \| \| ENSG00000226287 \| \| \| TRIM16L \| \| ENSG00000108448 \| \| \| OSGIN1 \| \| ENSG00000140961 \| \| \| NUMBL \| \| ENSG00000105245 \| \| \| HGD \| \| ENSG00000113924 \| \| \| TREML3P \| \| ENSG00000184106 \| \| \| AC245036.5 \| \| ENSG00000269271 \| \| \| AC113189.4 \| \| ENSG00000272884 \| \| \| OXCT1 \| ENSG00000083720 \| \| |
| --- | --- | --- | --- | --- | --- | --- | --- | --- | --- | --- | --- | --- | --- | --- | --- | --- | --- | --- | --- | --- | --- | --- | --- | --- | --- | --- | --- | --- | --- | --- | --- | --- | --- | --- | --- | --- | --- | --- | --- | --- | --- | --- | --- | --- | --- | --- | --- | --- | --- | --- | --- | --- | --- | --- | --- | --- | --- | --- | --- | --- | --- | --- | --- | --- | --- | --- | --- | --- | --- | --- | --- | --- | --- | --- | --- | --- | --- | --- | --- | --- | --- | --- | --- | --- | --- | --- | --- | --- | --- | --- | --- | --- | --- | --- | --- | --- | --- | --- | --- | --- | --- | --- | --- | --- | --- | --- | --- | --- | --- | --- | --- | --- | --- | --- | --- | --- | --- | --- | --- | --- | --- | --- | --- | --- | --- | --- | --- | --- | --- | --- | --- | --- | --- | --- | --- | --- | --- | --- | --- | --- | --- | --- | --- | --- | --- | --- | --- | --- | --- | --- | --- | --- | --- | --- | --- | --- | --- | --- | --- | --- | --- | --- | --- | --- | --- | --- | --- | --- | --- | --- | --- | --- | --- | --- | --- | --- | --- | --- | --- | --- | --- | --- | --- | --- | --- | --- | --- | --- | --- | --- | --- | --- | --- | --- | --- | --- | --- | --- | --- | --- | --- | --- | --- | --- | --- | --- | --- | --- | --- | --- | --- | --- | --- | --- | --- | --- | --- | --- | --- | --- | --- | --- | --- | --- | --- | --- | --- | --- | --- | --- | --- | --- | --- | --- | --- | --- | --- | --- | --- | --- | --- | --- | --- | --- | --- | --- | --- | --- | --- | --- | --- | --- | --- | --- | --- | --- | --- | --- | --- | --- | --- | --- | --- | --- | --- | --- | --- | --- | --- | --- | --- | --- | --- | --- | --- | --- | --- | --- | --- | --- | --- | --- | --- | --- | --- | --- | --- | --- | --- | --- | --- | --- | --- | --- | --- | --- | --- | --- | --- | --- | --- | --- | --- | --- | --- | --- | --- | --- | --- | --- | --- | --- | --- | --- | --- | --- | --- | --- | --- | --- | --- | --- | --- | --- | --- | --- | --- | --- | --- | --- | --- | --- | --- | --- | --- | --- | --- | --- | --- | --- | --- | --- | --- | --- | --- | --- | --- | --- | --- | --- | --- | --- | --- | --- | --- | --- | --- | --- | --- | --- | --- | --- | --- | --- | --- | --- | --- | --- | --- | --- | --- | --- | --- | --- | --- | --- | --- | --- | --- | --- | --- | --- | --- | --- | --- | --- | --- | --- | --- | --- | --- | --- | --- | --- | --- | --- | --- | --- | --- | --- | --- | --- | --- | --- | --- | --- | --- | --- | --- | --- | --- | --- | --- | --- | --- | --- | --- | --- | --- | --- | --- | --- | --- | --- | --- | --- | --- | --- | --- | --- | --- | --- | --- | --- | --- | --- | --- | --- | --- | --- | --- | --- | --- | --- | --- | --- | --- | --- | --- | --- | --- | --- | --- | --- | --- | --- | --- | --- | --- | --- | --- | --- | --- | --- | --- | --- | --- | --- | --- | --- | --- | --- | --- | --- | --- | --- | --- | --- | --- | --- | --- | --- | --- | --- | --- | --- | --- | --- | --- | --- | --- | --- | --- | --- | --- | --- | --- | --- | --- | --- | --- | --- | --- | --- | --- | --- | --- | --- | --- | --- | --- | --- | --- | --- | --- | --- | --- | --- | --- | --- | --- | --- | --- | --- | --- | --- | --- | --- | --- | --- | --- | --- | --- | --- | --- | --- | --- | --- | --- | --- | --- | --- | --- | --- | --- | --- | --- | --- | --- | --- | --- | --- | --- | --- | --- | --- | --- | --- | --- | --- | --- | --- | --- | --- | --- | --- | --- | --- | --- | --- | --- | --- | --- | --- | --- | --- | --- | --- | --- | --- | --- | --- | --- | --- | --- | --- | --- | --- | --- | --- | --- | --- | --- | --- | --- | --- | --- | --- | --- | --- | --- | --- | --- | --- | --- | --- | --- | --- | --- | --- | --- | --- | --- | --- | --- | --- | --- | --- | --- | --- | --- | --- | --- | --- | --- | --- | --- | --- | --- | --- | --- | --- | --- | --- | --- | --- | --- | --- | --- | --- | --- | --- | --- | --- | --- | --- | --- | --- | --- | --- | --- | --- | --- | --- | --- | --- | --- | --- | --- | --- | --- | --- | --- | --- | --- | --- | --- | --- | --- | --- | --- | --- | --- | --- | --- | --- | --- | --- | --- | --- | --- | --- | --- | --- | --- | --- | --- | --- | --- | --- | --- | --- | --- | --- | --- | --- | --- | --- | --- | --- | --- | --- | --- | --- | --- | --- | --- |

Listed genes satisfied the following criteria q<0.05; highlighted in yellow are those with a log-fold change of 1.5

**Additional table 2: Genes identified after incubation with MNPA with significant increased expression after Bonferroni correction**

| Gene name Gene ID | Gene name Gene ID | Gene name Gene ID |
| --- | --- | --- |
| \| H3F3AP4 \| ENSG00000235655 \| \| --- \| --- \| \| PRKN \| ENSG00000185345 \| \| ARSEP1 \| ENSG00000224060 \| \| MYO7B \| ENSG00000169994 \| \| SIGLEC6 \| ENSG00000105492 \| \| WDR49 \| ENSG00000174776 \| \| TENM3 \| ENSG00000218336 \| \| GLDN \| ENSG00000186417 \| \| GRIP1 \| ENSG00000155974 \| \| NEGR1 \| ENSG00000172260 \| \| LRMDA \| ENSG00000148655 \| \| CCDC26 \| ENSG00000229140 \| \| AL023693.1 \| ENSG00000224374 \| \| KCNQ5 \| ENSG00000185760 \| \| AL109914.1 \| ENSG00000229646 \| \| C2orf81 \| ENSG00000284308 \| \| CNTN4 \| ENSG00000144619 \| \| CSPG4 \| ENSG00000173546 \| \| LGALS12 \| ENSG00000133317 \| \| MS4A3 \| ENSG00000149516 \| \| KIT \| ENSG00000157404 \| \| MEGF6 \| ENSG00000162591 \| \| AC104232.1 \| ENSG00000254006 \| \| NREP \| ENSG00000134986 \| \| AC113133.1 \| ENSG00000253389 \| \| ANXA8 \| ENSG00000265190 \| \| SDK1 \| ENSG00000146555 \| \| TMEM231 \| ENSG00000205084 \| \| PDGFD \| ENSG00000170962 \| \| COL23A1 \| ENSG00000050767 \| \| RXFP1 \| ENSG00000171509 \| \| KCNA3 \| ENSG00000177272 \| \| PLB1 \| ENSG00000163803 \| \| TGM5 \| ENSG00000104055 \| \| TSPAN32 \| ENSG00000064201 \| \| DENND1A \| ENSG00000119522 \| \| CLSTN2 \| ENSG00000158258 \| \| MCOLN2 \| ENSG00000153898 \| \| PIF1 \| ENSG00000140451 \| \| DEPTOR \| ENSG00000155792 \| \| NCAM2 \| ENSG00000154654 \| \| TPM2 \| ENSG00000198467 \| \| KCNQ1 \| ENSG00000053918 \| \| HLA-F \| ENSG00000204642 \| \| CRABP1 \| ENSG00000166426 \| \| THBS4 \| ENSG00000113296 \| \| MATK \| ENSG00000007264 \| \| THSD7A \| ENSG00000005108 \| \| HSD11B1 \| ENSG00000117594 \| \| OXCT2 \| ENSG00000198754 \| \| ANKRD45 \| ENSG00000183831 \| \| MYL9 \| ENSG00000101335 \| \| LTC4S \| ENSG00000213316 \| \| COL27A1 \| ENSG00000196739 \| \| NTSR1 \| ENSG00000101188 \| \| ZFP36 \| ENSG00000128016 \| \| FBN2 \| ENSG00000138829 \| \| AC125603.2 \| ENSG00000257883 \| \| LINC00996 \| ENSG00000242258 \| \| JUNB \| ENSG00000171223 \| \| MANF \| ENSG00000145050 \| \| CLYBL \| ENSG00000125246 \| \| DANT2 \| ENSG00000235244 \| \| MLC1 \| ENSG00000100427 \| \| BCL2 \| ENSG00000171791 \| \| RASAL2 \| ENSG00000075391 \| \| PTGES3L \| ENSG00000267060 \| \| PTGES3L-AARSD1 \| ENSG00000108825 \| \| MEF2D \| ENSG00000116604 \| \| AC090796.1 \| ENSG00000253821 \| \| AFF2 \| ENSG00000155966 \| \| TGFBR3 \| ENSG00000069702 \| \| NID1 \| ENSG00000116962 \| \| SPNS3 \| ENSG00000182557 \| \| AC012651.1 \| ENSG00000258461 \| \| TSPOAP1-AS1 \| ENSG00000265148 \| \| ACPP \| ENSG00000014257 \| \| TESMIN \| ENSG00000132749 \| \| KIF20A \| ENSG00000112984 \| \| C20orf197 \| ENSG00000176659 \| \| DISC1 \| ENSG00000162946 \| \| MXD3 \| ENSG00000213347 \| \| PTMA \| ENSG00000187514 \| \| ANKRD36C \| ENSG00000174501 \| \| PLD4 \| ENSG00000166428 \| \| DCHS1 \| ENSG00000166341 \| \| PHACTR1 \| ENSG00000112137 \| \| MYBPH \| ENSG00000133055 \| \| LPL \| ENSG00000175445 \| \| VSIG4 \| ENSG00000155659 \| \| ATP8B3 \| ENSG00000130270 \| \| MYADM \| ENSG00000179820 \| \| PHACTR3 \| ENSG00000087495 \| \| PRKCB \| ENSG00000166501 \| \| Sep-05 \| ENSG00000184702 \| \| DIAPH2 \| ENSG00000147202 \| \| FAM163A \| ENSG00000143340 \| \| ACTB \| ENSG00000075624 \| \| ACTG1 \| ENSG00000184009 \| \| GAMT \| ENSG00000130005 \| \| CPM \| ENSG00000135678 \| \| NUP210 \| ENSG00000132182 \| \| FRAT1 \| ENSG00000165879 \| \| BAHCC1 \| ENSG00000266074 \| \| ARHGAP15 \| ENSG00000075884 \| \| AC092683.1 \| ENSG00000230606 \| \| H1FX \| ENSG00000184897 \| \| SLC17A9 \| ENSG00000101194 \| \| CD209 \| ENSG00000090659 \| \| CENPV \| ENSG00000166582 \| \| PTPRG \| ENSG00000144724 \| \| WDR54 \| ENSG00000005448 \| \| BIK \| ENSG00000100290 \| \| AC073957.3 \| ENSG00000273151 \| \| APBA2 \| ENSG00000034053 \| \| EXOC4 \| ENSG00000131558 \| \| MICAL1 \| ENSG00000135596 \| \| AL135999.1 \| ENSG00000258727 \| \| PSMB8-AS1 \| ENSG00000204261 \| \| SLC2A9 \| ENSG00000109667 \| \| CALR \| ENSG00000179218 \| | \| ZNF618 \| ENSG00000157657 \| \| --- \| --- \| \| SCFD2 \| ENSG00000184178 \| \| KCNMB1 \| ENSG00000145936 \| \| VIM \| ENSG00000026025 \| \| ALDH2 \| ENSG00000111275 \| \| ATP2B4 \| ENSG00000058668 \| \| NRGN \| ENSG00000154146 \| \| CELF2 \| ENSG00000048740 \| \| BARX1 \| ENSG00000131668 \| \| C1orf228 \| ENSG00000198520 \| \| EEF2K \| ENSG00000103319 \| \| SATB1 \| ENSG00000182568 \| \| TNN \| ENSG00000120332 \| \| RABGAP1L \| ENSG00000152061 \| \| AC060780.1 \| ENSG00000267002 \| \| GSN \| ENSG00000148180 \| \| CABIN1 \| ENSG00000099991 \| \| PDIA4 \| ENSG00000155660 \| \| ARHGEF39 \| ENSG00000137135 \| \| DNAAF2 \| ENSG00000165506 \| \| MS4A7 \| ENSG00000166927 \| \| FSCN1 \| ENSG00000075618 \| \| RSU1 \| ENSG00000148484 \| \| ANAPC15 \| ENSG00000110200 \| \| TBC1D5 \| ENSG00000131374 \| \| YBX3 \| ENSG00000060138 \| \| BUB1 \| ENSG00000169679 \| \| PDIA5 \| ENSG00000065485 \| \| NLRP3 \| ENSG00000162711 \| \| IGFBP2 \| ENSG00000115457 \| \| MTHFD1L \| ENSG00000120254 \| \| NAGA \| ENSG00000198951 \| \| CD44 \| ENSG00000026508 \| \| CNN2 \| ENSG00000064666 \| \| HSP90B1 \| ENSG00000166598 \| \| PARD3 \| ENSG00000148498 \| \| PRKCA \| ENSG00000154229 \| \| GPSM2 \| ENSG00000121957 \| \| SLC22A31 \| ENSG00000259803 \| \| FLOT2 \| ENSG00000132589 \| \| CMSS1 \| ENSG00000184220 \| \| CCNF \| ENSG00000162063 \| \| TBC1D22A \| ENSG00000054611 \| \| PLTP \| ENSG00000100979 \| \| PKP4 \| ENSG00000144283 \| \| CRELD2 \| ENSG00000184164 \| \| FLT3 \| ENSG00000122025 \| \| ASS1 \| ENSG00000130707 \| \| C14orf159 \| ENSG00000133943 \| \| CCNB1 \| ENSG00000134057 \| \| SEL1L3 \| ENSG00000091490 \| \| UBB \| ENSG00000170315 \| \| LIMA1 \| ENSG00000050405 \| \| SH3KBP1 \| ENSG00000147010 \| \| CDCA7 \| ENSG00000144354 \| \| ESYT2 \| ENSG00000117868 \| \| CCDC144NL-AS1 \| ENSG00000233098 \| \| ALCAM \| ENSG00000170017 \| \| PPIB \| ENSG00000166794 \| \| TAGLN2 \| ENSG00000158710 \| \| PARVG \| ENSG00000138964 \| \| CDH23 \| ENSG00000107736 \| \| CSF3R \| ENSG00000119535 \| \| FAF1 \| ENSG00000185104 \| \| EHD4 \| ENSG00000103966 \| \| ITGA4 \| ENSG00000115232 \| \| SCMH1 \| ENSG00000010803 \| \| TUBB4B \| ENSG00000188229 \| \| FAM60A \| ENSG00000139146 \| \| HLA-B \| ENSG00000234745 \| \| NEU1 \| ENSG00000204386 \| \| PSMA1 \| ENSG00000129084 \| \| PSMC2 \| ENSG00000161057 \| \| UBE2A \| ENSG00000077721 \| \| CEBPG \| ENSG00000153879 \| \| ZNF684 \| ENSG00000117010 \| \| ALAS1 \| ENSG00000023330 \| \| TPRA1 \| ENSG00000163870 \| \| HTATIP2 \| ENSG00000109854 \| \| PSMA3 \| ENSG00000100567 \| \| ARMCX3 \| ENSG00000102401 \| \| ZUFSP \| ENSG00000153975 \| \| SLC5A3 \| ENSG00000198743 \| \| HMCES \| ENSG00000183624 \| \| GLRX2 \| ENSG00000023572 \| \| PRNP \| ENSG00000171867 \| \| WASHC2C \| ENSG00000172661 \| \| HMGCL \| ENSG00000117305 \| \| TOM1 \| ENSG00000100284 \| \| SLA \| ENSG00000155926 \| \| MED10 \| ENSG00000133398 \| \| RHOC \| ENSG00000155366 \| \| HINT3 \| ENSG00000111911 \| \| ASF1A \| ENSG00000111875 \| \| CLN5 \| ENSG00000102805 \| \| LGALS3 \| ENSG00000131981 \| \| ETS1 \| ENSG00000134954 \| \| TRAFD1 \| ENSG00000135148 \| \| RECQL \| ENSG00000004700 \| \| RHBDD2 \| ENSG00000005486 \| \| SPHK1 \| ENSG00000176170 \| \| IFI6 \| ENSG00000126709 \| \| TMBIM1 \| ENSG00000135926 \| \| UPF3B \| ENSG00000125351 \| \| DTX3L \| ENSG00000163840 \| \| EMP3 \| ENSG00000142227 \| \| SAMD9 \| ENSG00000205413 \| \| HEXIM1 \| ENSG00000186834 \| \| C19orf66 \| ENSG00000130813 \| \| CTSO \| ENSG00000256043 \| \| RIPK1 \| ENSG00000137275 \| \| EMC3 \| ENSG00000125037 \| \| PPARG \| ENSG00000132170 \| \| SOWAHC \| ENSG00000198142 \| \| GSR \| ENSG00000104687 \| \| NPL \| ENSG00000135838 \| \| PRDX1 \| ENSG00000117450 \| \| HEBP1 \| ENSG00000013583 \| \| KIF21A \| ENSG00000139116 \| \| CASP3 \| ENSG00000164305 \| \| RTP4 \| ENSG00000136514 \| \|  \|  \| | \| CD55 \| ENSG00000196352 \| \| --- \| --- \| \| ADAMDEC1 \| ENSG00000134028 \| \| TNFAIP2 \| ENSG00000185215 \| \| CTSL \| ENSG00000135047 \| \| LPAR1 \| ENSG00000198121 \| \| FILIP1L \| ENSG00000168386 \| \| CARD19 \| ENSG00000165233 \| \| F11R \| ENSG00000158769 \| \| FAM177A1 \| ENSG00000151327 \| \| DFNA5 \| ENSG00000105928 \| \| PEX13 \| ENSG00000162928 \| \| ZNF85 \| ENSG00000105750 \| \| MKNK2 \| ENSG00000099875 \| \| ARRDC4 \| ENSG00000140450 \| \| MCOLN1 \| ENSG00000090674 \| \| MTHFS \| ENSG00000136371 \| \| PGD \| ENSG00000142657 \| \| LFNG \| ENSG00000106003 \| \| NINJ1 \| ENSG00000131669 \| \| CYP2R1 \| ENSG00000186104 \| \| DNAJB2 \| ENSG00000135924 \| \| GLRX \| ENSG00000173221 \| \| TXNRD1 \| ENSG00000198431 \| \| DYNLT3 \| ENSG00000165169 \| \| SERTAD1 \| ENSG00000197019 \| \| GRIN3A \| ENSG00000198785 \| \| BLOC1S2 \| ENSG00000196072 \| \| PEA15 \| ENSG00000162734 \| \| DYNLT1 \| ENSG00000146425 \| \| CASP7 \| ENSG00000165806 \| \| GADD45A \| ENSG00000116717 \| \| FTL \| ENSG00000087086 \| \| IFI16 \| ENSG00000163565 \| \| FZD5 \| ENSG00000163251 \| \| APOL6 \| ENSG00000221963 \| \| CREM \| ENSG00000095794 \| \| NMT2 \| ENSG00000152465 \| \| PARP14 \| ENSG00000173193 \| \| KREMEN1 \| ENSG00000183762 \| \| NQO2 \| ENSG00000124588 \| \| ZNF703 \| ENSG00000183779 \| \| PARP9 \| ENSG00000138496 \| \| OSBPL11 \| ENSG00000144909 \| \| CD300LB \| ENSG00000178789 \| \| PLEKHF1 \| ENSG00000166289 \| \| KIAA1522 \| ENSG00000162522 \| \| GOT1 \| ENSG00000120053 \| \| IRF1 \| ENSG00000125347 \| \| OCIAD2 \| ENSG00000145247 \| \| CPEB4 \| ENSG00000113742 \| \| MARCKSL1 \| ENSG00000175130 \| \| APOL2 \| ENSG00000128335 \| \| MSC-AS1 \| ENSG00000235531 \| \| STXBP5-AS1 \| ENSG00000233452 \| \| FUCA1 \| ENSG00000179163 \| \| LPXN \| ENSG00000110031 \| \| L2HGDH \| ENSG00000087299 \| \| ULBP1 \| ENSG00000111981 \| \| SNHG1 \| ENSG00000255717 \| \| MPZ \| ENSG00000158887 \| \| RIT1 \| ENSG00000143622 \| \| TP53INP1 \| ENSG00000164938 \| \| FAM111B \| ENSG00000189057 \| \| FAM109A \| ENSG00000198324 \| \| EPHX1 \| ENSG00000143819 \| \| CLGN \| ENSG00000153132 \| \| LACC1 \| ENSG00000179630 \| \| PSMC3IP \| ENSG00000131470 \| \| MPZL3 \| ENSG00000160588 \| \| IFIT5 \| ENSG00000152778 \| \| LILRA5 \| ENSG00000187116 \| \| ITPKA \| ENSG00000137825 \| \| PIR \| ENSG00000087842 \| \| ABCA1 \| ENSG00000165029 \| \| TMEM255A \| ENSG00000125355 \| \| G6PD \| ENSG00000160211 \| \| GTPBP2 \| ENSG00000172432 \| \| TNFRSF12A \| ENSG00000006327 \| \| ZNF697 \| ENSG00000143067 \| \| LY96 \| ENSG00000154589 \| \| AC254633.1 \| ENSG00000272482 \| \| B4GALNT1 \| ENSG00000135454 \| \| SRXN1 \| ENSG00000271303 \| \| MSC \| ENSG00000178860 \| \| GCLM \| ENSG00000023909 \| \| SLAMF7 \| ENSG00000026751 \| \| ME1 \| ENSG00000065833 \| \| ABCG1 \| ENSG00000160179 \| \| CYP1B1 \| ENSG00000138061 \| \| FTH1 \| ENSG00000167996 \| \| TRIM16L \| ENSG00000108448 \| \| OSGIN1 \| ENSG00000140961 \| \| HGD \| ENSG00000113924 \| \| SUCNR1 \| ENSG00000198829 \| \| TRIB3 \| ENSG00000101255 \| \| SLFN5 \| ENSG00000166750 \| \| CRTAM \| ENSG00000109943 \| \| NQO1 \| ENSG00000181019 \| \| SEMA6B \| ENSG00000167680 \| \| INHBE \| ENSG00000139269 \| \| DLGAP1-AS2 \| ENSG00000262001 \| \| CLU \| ENSG00000120885 \| \| EFNB2 \| ENSG00000125266 \| \| HTRA3 \| ENSG00000170801 \| \| SPTA1 \| ENSG00000163554 \| \| HMOX1 \| ENSG00000100292 \| \| LUCAT1 \| ENSG00000248323 \| \| OLAH \| ENSG00000152463 \| \| TMEM140 \| ENSG00000146859 \| \| NMRAL2P \| ENSG00000171658 \| \| U62317.1 \| ENSG00000226738 \| \| KLHDC7B \| ENSG00000130487 \| \| AL596330.1 \| ENSG00000229400 \| \| TREML3P \| ENSG00000184106 \| \| SGCG \| ENSG00000102683 \| \| NEUROD4 \| ENSG00000123307 \| \| TREML4 \| ENSG00000188056 \| |

Listed genes satisfied the following criteria q<0.05; highlighted in yellow are those with a log-fold change of 1.5

**Table S3.** **Primer sequences used in qPCR analysis**

<https://www.ncbi.nlm.nih.gov/pmc/articles/PMC2851245/>

| **Gene** | **Primer sequence forward** | **Primer sequence reverse** |
| --- | --- | --- |
| 18S | CCGCAGCTAGGAATAATGGAATA | TCTAGCGGCGCAATACGAAT |
| IL-6 | GTAGCCGCCCCACACAGA | CATGTCTCCTTTCTCAGGGCTG |
| IL-8 | ATAAAGACATACTCCAAACCTTTCCAC | AAGCTTTACAATAATTTCTGTGTTGGC |
| TNFa | CCCAGGGACCTCTCTCTAATCA | GCTTGAGGGTTTGCTACAACATG |
